# Resources for Human Health from the Plant Kingdom: The Potential Role of the Flavonoid Apigenin in Cancer Counteraction

**DOI:** 10.3390/ijms25010251

**Published:** 2023-12-23

**Authors:** Laura Fossatelli, Zaira Maroccia, Carla Fiorentini, Massimo Bonucci

**Affiliations:** 1 Association for Research on Integrative Oncology Therapies (ARTOI) Foundation, Via Ludovico Micara 73, 00165 Rome, Italy; laura.fossatelli@gmail.com (L.F.); maxbonucci@artoi.it (M.B.); 2 Department of Cardiovascular, Endocrine-Metabolic Diseases and Aging, Istituto Superiore di Sanità, Viale Regina Elena 299, 00161 Rome, Italy; zaira.maroccia@iss.it

**Keywords:** apigenin, flavonoids, neoplasms, bioavailability, combined modality therapies

## Abstract

Apigenin is one of the most widespread flavonoids in the plant kingdom. For centuries, apigenin-containing plant preparations have been used in traditional medicines to treat diseases that have an inflammatory and/or degenerative component. In the 1980s, apigenin was proposed to interfere with the process of carcinogenesis. Since then, more and more evidence has demonstrated its anticancer efficacy, both in vitro and in vivo. Apigenin has been shown to target signaling pathways involved in the development and progression of cancer, such as PI3K/Akt/mTOR, MAPK/ERK, JAK/STAT, NF-κB, and Wnt/β-catenin pathways, and to modulate different hallmarks of cancer, such as cell proliferation, metastasis, apoptosis, invasion, and cell migration. Furthermore, apigenin modulates PD1/PD-L1 expression in cancer/T killer cells and regulates the percentage of T killer and T regulatory cells. Recently, apigenin has been studied for its synergic and additive effects when combined with chemotherapy, minimizing the side effects. Unfortunately, its low bioavailability and high permeability limit its therapeutic applications. Based on micro- and nanoformulations that enhance the physical stability and drug-loading capacity of apigenin and increase the bioavailability of apigenin, novel drug-delivery systems have been investigated to improve its solubility.

## 1. Introduction

In countries with higher economic development levels, cancer is the second leading cause of death behind cardiovascular disease. According to recent data, in Italy, 415,269 new diagnoses of cancer occurred, with 174,759 cancer deaths (96,579 in men and 78,180 in women) [1].

Cancer is a disease that originates and evolves through genetic and epigenetic modifications involving genes that control cell cycle, adhesion, motility, differentiation, and apoptosis [2]. Human cancers develop as products of multistep processes in which cells acquire functional capabilities that are crucial for their ability to induce malignant tumors [2,3]. Such capabilities are called “Hallmarks of Cancer” and consist of sustaining proliferative signaling, evading growth suppressors, resisting cell death, enabling replicative immortality, inducing/accessing vasculature, activating invasion and metastasis, reprogramming cellular metabolism, and avoiding immune destruction [4,5,6]. These hallmarks are further enriched by two “enabling characteristics” involved in tumor growth and progression: tumor-promoting inflammation and genome instability and mutation. The tumor microenvironment plays an integral role in tumorigenesis and malignant progression, too [7,8]. Emerging hallmarks also include unlocking phenotypic plasticity, a capability that enables various disruptions of cellular differentiation and appears to be operative in multiple cancer types during primary tumor formation, malignant progression, and/or response to therapy. Another emerging hallmark is non-mutational epigenetic reprogramming, since growing evidence shows that gene-regulatory circuits and networks in tumors can be governed by numerous corrupted and added mechanisms, independent of genome instability and gene mutation. Moreover, the polymorphic microbiomes resident in the colon, other mucosa, and connected organs, or in tumors themselves, can diversely influence cancer development, progression, and response to therapy. Finally, an increasing body of evidence reveals that, in certain contexts, senescent cells variously stimulate tumor development and malignant progression [5].

Throughout centuries, natural compounds have been used to treat and prevent diseases [9,10], thereby creating new knowledge of the potential of natural agents. Almost 47% of the anticancer drugs available on the market today are derivatives of natural products [11]. Considering natural compounds, polyphenols (flavonoids, stilbenoids, lignans, polyphenolic acids, and other polyphenols) display many anticarcinogenic properties and, in addition, they can modulate immune system responses and protect normal cells against damage from free radicals [12]. A plethora of studies have documented these anticancer effects: noteworthy examples include anthocyanins from blueberries [13], epigallocatechin gallate from green tea [14], resveratrol from red wine [15], isoflavones from soy [16], and curcumin from *Curcuma longa* [14,17]. Among the over 6000 different flavonoids, quercetin, kaempferol, myricetin, luteolin, and apigenin are the five most ubiquitous plant flavonoids [18,19].

Apigenin, above all, has gained attention among researchers, partly due to its low toxicity and multiple beneficial bioactivities [18]. Apigenin is one of the most widespread flavonoids in the plant kingdom and one of the most studied phenolic compounds [20]. For centuries, apigenin-containing plant preparations have been used as traditional medicines to treat diseases with an inflammatory and/or degenerative component, such as asthma, insomnia, neuralgia, Parkinson’s disease, and shingles [21,22,23,24]. At the end of the 1950s, apigenin gained scientific interest, primarily due to its modulation of histamine release and bronchodilator properties [21]. In the 1980s, apigenin was proposed to interfere with the process of carcinogenesis. Since then, more and more evidence has suggested its power as an adjuvant chemotherapeutic agent for cancer therapy, both in in vitro and in vivo models [20].

## 2. General Aspects of Apigenin

### 2.1. Natural Sources

Of all the flavonoids, apigenin is the most widely distributed in the plant kingdom, found in numerous types of vegetables, fruits, herbs, and spices [21]. Its name is derived from the *Apium* genus in *Apiaceae* or *Umbelliferae*, to which parsley and celery belong, species constituting its major sources in nature. In a 100 g edible portion, parsley (*Petroselinum crispum*) contains 215.46 mg if it is consumed fresh and 4503.50 mg if it is consumed dried, while fresh celery (*Apium graveolens*) contains 19.10 mg and its seeds contain 78.65 mg [25]. Other sources of high apigenin content include Chinese celery, celeriac, white and red sorghum, onions, tea, maize, oranges, wheat sprouts, oregano, thyme, peppermint, rosemary, sage, artichoke, juniper berries, and kumquat [25].

Apigenin is the most prevalent flavonoid in the dried flowers of *Matricaria chamomilla*: infusions of chamomile contain maximum concentrations of apigenin ranging from 0.8 to 1.2%. In nature, apigenin also exists as a dimer, biapigenin, mainly isolated from the buds and flowers of *Hypericum perforatum* [26]. Some other plants from which apigenin and its derivatives are obtained are listed in the Supplementary Table S1.

Moreover, the cannabis plant produces a considerable number of flavonoids, and their distribution varies in the plant, focusing mainly on flowers, leaves, and stems. Their total content reaches about 2.5% of the dry weight of flowers and leaves, while it is almost non-existent in seeds and roots. Apigenin is one of the flavonoidal molecules that (together with kaempferol, quercetin, vitexin, and isovitexin, luteolin, and orientina) modifies the activity of Δ-9-tetrahydrocannabinol through a mechanism shared with cannabidiol and some terpenes [27,28,29].

### 2.2. Chemistry and Biological Activity

**Figure 1:**
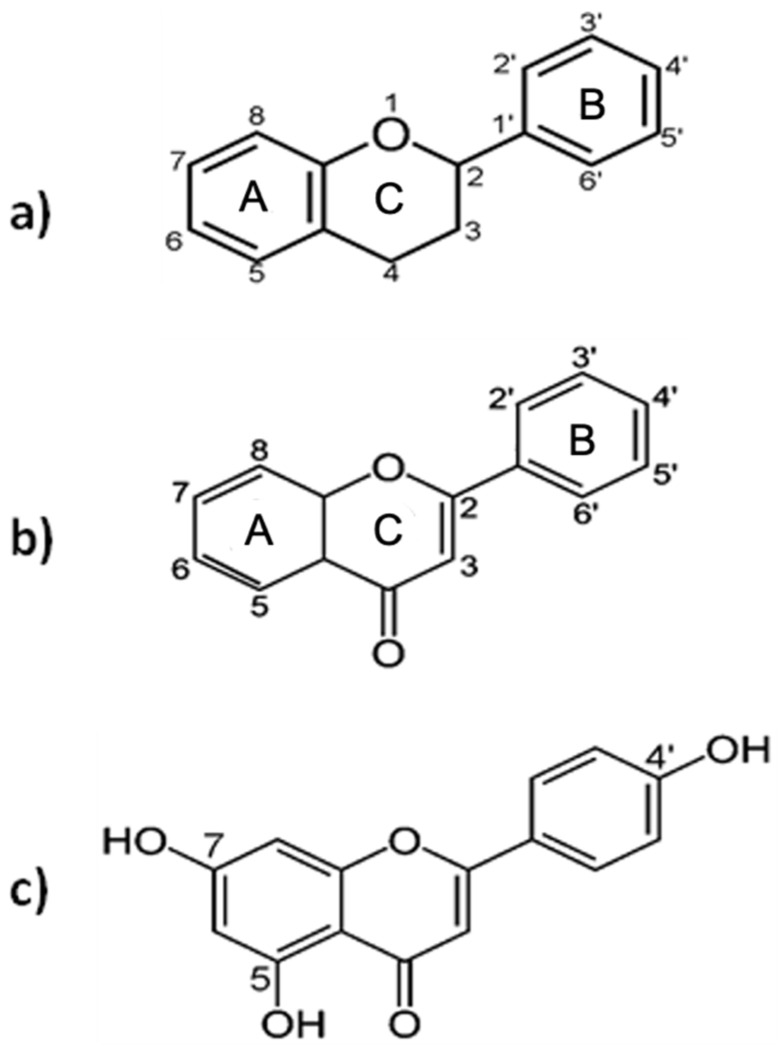
(**a**) The flavonoid basic structure; (**b**) flavone; (**c**) apigenin. The two benzene rings (A and B) are connected by a three-carbon linking chain that, with a benzenic A ring, forms the heterocyclic C ring containing oxygen.

Flavonoids are plant secondary metabolites that share a common carbon skeleton of diphenylpropane, containing two benzene rings (A and B) connected by a three-carbon linking chain that forms a heterocyclic ring containing oxygen, the C ring, with a benzenic A ring (Figure 1a). In most cases, the B ring is attached to position 2 of the C ring, but it can also bind in position 3 or 4. Flavonoids in which the B ring is linked in position 3 are called isoflavones and those in which the B ring is linked in position 4 are called neoflavonoids. Those in which the B ring is linked in position 2 can be further subdivided into 6 subgroups, based on the structural features of the C ring: flavones, flavonols, flavanones, flavanonols, flavanols or catechins, anthocyanins, and chalcones. Flavones differ from other flavonoids in that they have a double bond between C2 and C3; there is no substitution at the C3 position, and they are oxidized at the C4 position (Figure 1b) [30].

The compound apigenin (4′,5,7-trihydroxyflavone) is a natural flavone with the molecular formula C_15_H_10_O_5_ and a molecular weight of 270.24 g/mol [31]. It has three hydroxyl groups, with the first and second in the C5 and C7 positions, and the third at C4′ of the B ring (Figure 1c). It is a yellow crystalline powder that is insoluble in water and soluble in dimethyl sulfoxide and hot ethanol. According to the Biopharmaceutics Classification System, apigenin is categorized as a Class II drug, whose characteristics are low solubility and high permeability: its absorption is therefore limited by its slow dissolution in the gastrointestinal tract [32]. In nature, apigenin mainly exists in O- and C- glycosidic forms and occasionally as aglycone. The most common apigenin glycosides are apigenin-7-O-apiosylglucoside (apiin), apigenin-7-O-glucoside, apigenin-8-C-glucoside (vitexin), and apigenin-6-C-glucoside (isovitexin). Flavonoids are characterized by a wide range of biological activities, such as antioxidant, anti-inflammatory, antimutagenic, and antiviral activities, demonstrated by numerous in vitro and in vivo studies [33,34]. Moreover, flavonoids can reduce plasma low-density lipoprotein levels, inhibit platelet aggregation, and reduce cell proliferation [35,36,37]. In the context of systemic inflammation and the reactivations of the Zoster and Epstein–Barr virus (EBV), the results of the interference of apigenin in viral reactivations are highlighted [38]. The EBV, a member of the γ-herpesviruses, infecting the majority of the world’s human population, plays a causal role in infectious mononucleosis, hairy leukoplakia, and post-transplant lymphoproliferative disorders, and is highly associated with several human malignancies, including Burkitt’s lymphoma and nasopharyngeal carcinoma (NPC). The EBV primarily infects circulating human B cells and is kept in a latent state. The lytic reactivation of the EBV plays an important role in various human malignancies, being highly correlated with cancer progression, poor prognosis, and tumor recurrence of NPC, peculiarly [39]. The ability of apigenin to inhibit the lytic induction of the EBV, reactivation in the lytic cycle, and virion production by EBV-positive NPC cells, appears to be beneficial for cancer prevention [40,41]. Wu et al. [38] have provided new insights into the biological application of apigenin for anti-EBV therapy. The detection of lytic proteins using a Western blot analysis showed that the EBV could normally express lytic proteins. However, the protein expression was gradually repressed after apigenin treatment, demonstrating that apigenin can inhibit EBV reactivation and repress EBV lytic protein expressions, acting as a potential dietary compound for preventing EBV reactivation [38].

**Figure 2:**
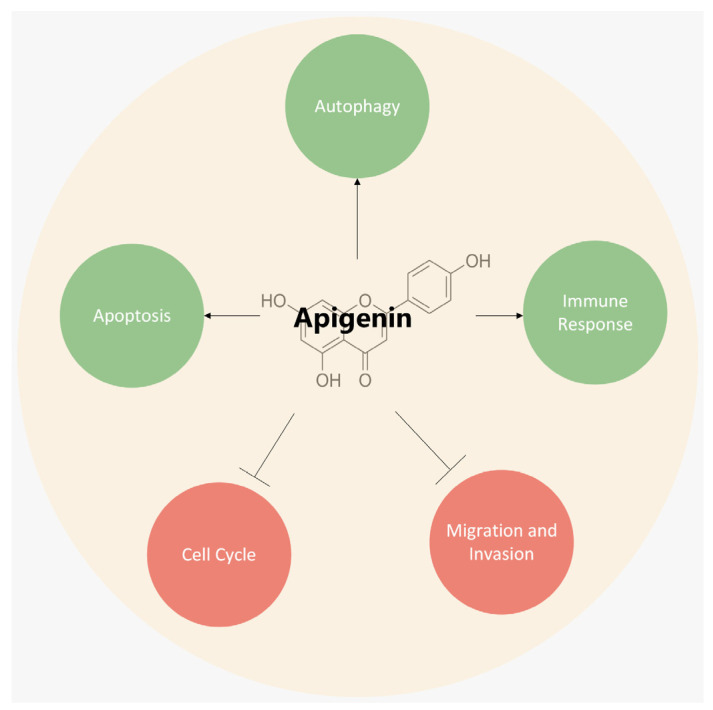
Apigenin (APG) induces apoptosis and autophagy, stimulates immune response, and inhibits cell-cycle progress and migration, and invasion by modulating signaling pathways involved in the development and progression of cancer [1].

Apigenin promotes different anti-inflammatory pathways, including the p38/MAPK and PI3K/Akt pathway, prevents the IKB degradation and nuclear translocation of the NF-κB, and reduces COX-2 activity [42,43]. Apigenin enhances the expression of anti-oxidant enzymes such as GSH-synthase, catalase, and superoxide dismutase to counteract cellular oxidative stress [35]. Furthermore, apigenin has been shown to inhibit, in vitro, rat brain monoamine oxidases and to have potential activity against depression and neurodegenerative diseases, such as Parkinson’s and Alzheimer’s disease [23,35]. Apigenin has also demonstrated anti-diabetic properties thanks to its capacity to inhibit glucosidase activity, increase the secretion of insulin, and interact with and neutralize reactive oxygen species (ROS) in the cell, contributing to the prevention of diabetic complications [35,44]. Studies carried out over the years have suggested that apigenin can be a therapeutic agent against different types of cancers. It has the ability, in vitro and in vivo, to modulate different hallmarks of cancer, such as cell proliferation, metastasis, apoptosis, invasion, and cell migration, and it can also stimulate an immune response [20,45] (Figure 2).

## 3. Mechanisms Underlying the Anticancer Role of Apigenin

In the 1980s, apigenin was indicated as a factor capable of interfering with the carcinogenesis process [46,47]. Since then, increasing evidence has proved the anticancer efficacy of apigenin, an activity underlined by several in vitro and in vivo mechanisms, including those listed below.

### 3.1. Induction of Apoptosis

Apigenin has been demonstrated in vitro and in vivo to be an effective agent for triggering apoptosis, via either the intrinsic or extrinsic pathway in many different human cancer cells [20,43,48]. Dysregulated apoptosis is a hallmark of cancer, being associated with unchecked cell proliferation, development, and the progression of cancer and cancer resistance to drug therapies. Thus, triggering cancer cell apoptosis by targeting apoptotic pathways is one of the major modes of action of chemotherapy drugs.

A study on the effects of a combined treatment with 5-fluorouracil (5-FU) and apigenin has shown cell growth inhibition and apoptosis induction via the downregulation of ErbB2 expression and Akt signaling in human breast cancer cells [49]. Similarly, apigenin enhances the anticancer effect of 5-FU against hepatocellular carcinoma cells in vitro and in vivo by promoting ROS production, mitochondrial membrane disruption, and apoptosis [49]. In mice with prostate cancer xenografts, the oral intake of apigenin at doses of 20 and 50 μg/mouse/day over 8 weeks results in a marked reduction in tumor growth and in a significant decrease in Bcl-2 expression with a concomitant increase in Bax [50].

**Figure 3:**
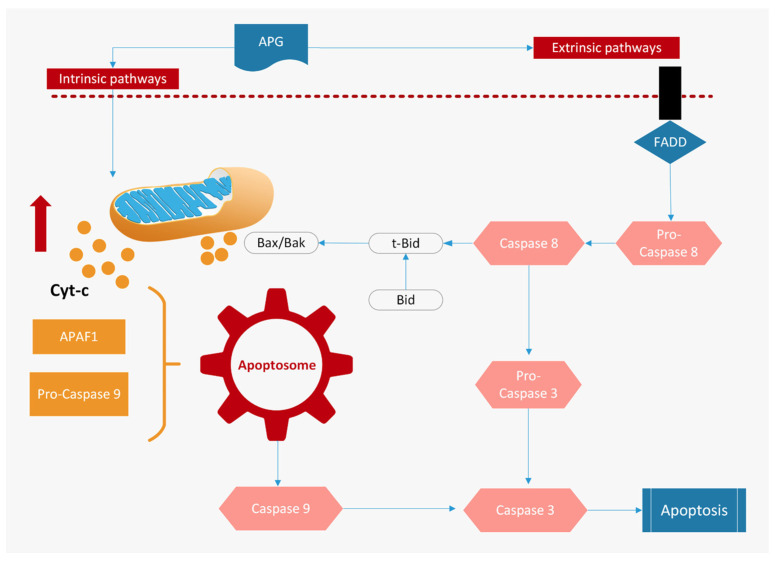
Apigenin (APG) can modulate both intrinsic and extrinsic apoptotic pathways [1]. In the intrinsic pathway, apigenin changes mitochondrial membrane potential and causes the release of cytochrome C (cyt-C) in the cytoplasm, where it binds to Apaf-1 to form the apoptosome, which in turn activates caspase 9, initiating the caspase cascade. In the extrinsic pathway, apigenin upregulates the expression of Fas, TRAIL, and TNF-ligands, and of the adaptor proteins, such as FADD, which recruits caspase-8. Furthermore, apigenin modulates the expression of Bcl-2, Bax, STAT-3, and Akt proteins.

A study of colon cancer cell lines has demonstrated that apigenin suppresses the expression of anti-apoptotic proteins and inhibits the phosphorylation of STAT3 [51] by the regulation of thymidylate synthetase [52]. Furthermore, in primary effusion lymphoma cell lines, apigenin upregulates the release of cytochrome C from mitochondria and downregulates the expression of the apoptosis protein inhibitor [53] (Figure 3).

### 3.2. Inhibition of Cell-Cycle Progressions

Apigenin causes cell-cycle arrest at various phases and modulates the expression of different cyclin-dependent kinases (CDKs) and other genes that regulate cell-cycle processes [37]. The cell cycle is an ordered series of events finely regulated in time and space by a set of proteins and enzymes that form the cell-cycle control system. At the heart of this cell-cycle control system, there are the cyclin-dependent kinases whose activity depends on an association with regulatory sub-units called cyclins. In eukaryotic cells, there are several points in the cell cycle, called checkpoints, at which the cycle can be arrested if previous events have not been completed or when damage to the genome or spindle is detected.

**Figure 4:**
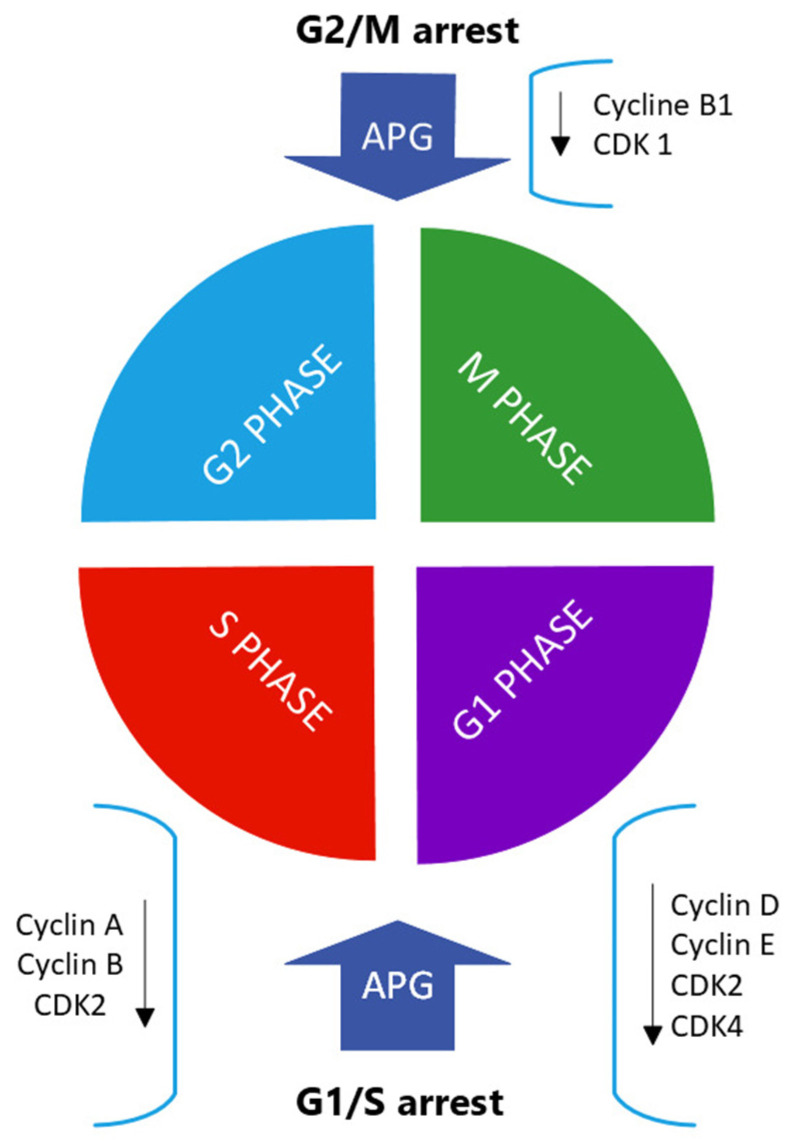
Apigenin (APG) induces cell-cycle arrest at various stages, such as G1/S and G2/M [1] and modulates the expression of CDKs and genes that control cell-cycle progress.

Cancer cells are characterized by mutations or alterations in regulatory proteins that induce defects in these checkpoint mechanisms [54,55]. Such defects very likely contribute to neoplastic transformation and progression by coupling genetic instability with resistance to apoptotic cell death and allowing these cells to replicate and divide abnormally. In HCT116 human colon cancer cells, apigenin inhibited cell proliferation by inducing G2/M phase arrest. This flavone also suppressed the expression of both cyclin B1 and its activating partners Cdc2 and Cdc25c, whereas the expression of cell-cycle inhibitors, such as p53 and p21, was increased after apigenin treatment [56]. In the MDA-MB-231 breast cancer cell line, apigenin suppressed the expression of cyclin A, cyclin B, and -1 (CDK1), which control the G2-to-M phase transition in the cell cycle [54]. In a tongue oral cancer-derived cell line, apigenin treatment resulted in cell-cycle arrest at both G0/G1 and G2/M checkpoints and in the decreased expression of cyclin D1 and E, and the inactivation of CDK1 [57]. In human prostate carcinoma cells, apigenin treatment resulted in G1 arrest and in a marked decrease in the protein expression of cyclin D1, -D2, and -E and their activating partner CDK-2, -4, and -6 [58] (Figure 4).

### 3.3. Induction of Autophagy

Apigenin-induced autophagy was first detected in erythroleukemia TF1 cells and, since then, more evidence has demonstrated that autophagy could be triggered by apigenin [59]. Autophagy is a highly conserved catabolic pathway that degrades cytoplasmic components, such as long-lived proteins and organelles, through lysosomes [60]. It is a process triggered by adverse environmental conditions, such as starvation, growth factor deprivation, pathogen infection, oxidative stress, and hormonal signaling.

The relationship between autophagy and cancer is complex and contradictory [61]. Autophagy, in fact, can act as a tumor suppressor by reducing mutagenesis or other damage caused by reactive oxygen species by the removal of damaged mitochondria and other organelles and limiting tumor cell growth. On the other hand, it allows cancer cells to respond to changing environmental conditions, such as nutrient deprivation, and stops them from dying by inhibiting apoptosis with a consequent resistance to therapies [48,62,63].

Apigenin can simultaneously induce both apoptosis and autophagy in human colon cancer HCT116 cells [56] and, similarly, in human breast cancer T47D and MDA-MB-231 cells [64]. Further studies have revealed that apigenin-induced apoptosis is significantly enhanced during treatment with the 3-methyladenine autophagy inhibitor. These findings show that autophagy triggered by apigenin performs a tumor-protective role in apigenin-caused cytotoxicity [56,64].

A study carried out on hepatocellular carcinoma HepG2 cells has shown that a combination of autophagy inhibitors and apigenin inhibits cell proliferation and induces autophagy, significantly enhancing the anticancer effect of apigenin [65].

### 3.4. Inhibition of Cell Migration and Invasion

Apigenin has been shown to inhibit cancer cell migration and invasion in several in vitro and in vivo studies, as already reviewed [20,66].

It has been demonstrated that apigenin suppresses the proliferation and inhibits the migration and invasive potential of DU145 prostate cancer cells in a dose- and time-dependent manner, which was associated with epithelial–mesenchymal transition (EMT) [67]. Also, 40 μM of apigenin on A375 and C8161 melanoma cell lines remarkably prevented cell migration and invasion by impacting the Akt/mTOR pathway [68]. Similarly, in the human A549 lung cancer cell line, apigenin arrests Akt phosphorylation and targets the PI3K/Akt signaling pathway, leading to anti-migration and anti-invasion effects [59]. In human A549 cells, apigenin can also downregulate type I collagen, vimentin, MMP-8, and VEGF, leading to a decrease in cell migration [37].

**Figure 5:**
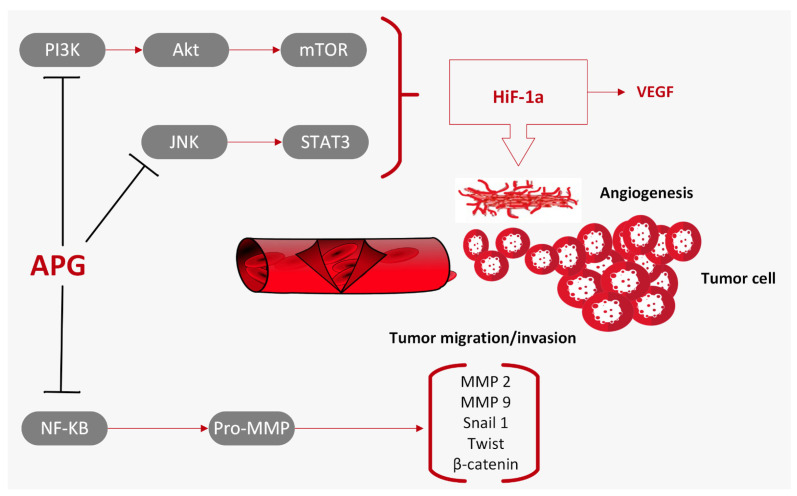
Apigenin (APG) inhibits metastasis and angiogenesis by modulating signaling pathways (MAPK, Akt, NF-κB) and the expression of their target genes (MMPs, snail, twist, β-catenin) associated with epithelial–mesenchymal transition [1]. Furthermore, APG downregulates HiF-1α and VEGF/VEGFR in the tumor environment.

Other studies have shown the ability of apigenin to inhibit STAT3 phosphorylation, its nuclear transportation and transcriptional activity, and to downregulate the expression of different genes involved in cell migration and invasion, such as MMP-2, MMP-9, VEGF, and Twist1 [37,69] (Figure 5).

### 3.5. Intracellular Signal Pathways Modulated by Apigenin

Apigenin has been reported to target multiple signaling pathways, such as the PI3K/Akt/mTOR, MAPK/ERK, JAK/STAT, NF-κB, and Wnt/β-catenin pathways, involved in the development and progression of cancer [20].

#### 3.5.1. Apigenin and the PI3K/Akt/mTOR Pathway

The PI3K/Akt/mTOR pathway is an intracellular signaling pathway involved in various cellular processes, such as cell growth, proliferation, and metabolism [58]. The hyperactivation of the PI3K/Akt/mTOR pathway is associated with drug resistance and cancer progression [70].

Apigenin acts on this signaling pathway in several ways. In different cell types, apigenin inhibits Akt function by directly suppressing PI3K activity by blocking the ATP-binding site of PI3K and subsequently inhibiting Akt kinase activity [71]. In breast cancer cells, it induces FOXO3a expression by inhibiting PI3K/Akt and, consequently, upregulates the expression of tumor suppressor proteins, such as the p21, CIP/WAF, and KIP1, thereby resulting in cell-cycle arrest and apoptosis [72]. In adriamycin-induced cardiotoxicity, apigenin actives the PI3K/Akt/mTOR pathway by upregulating the phosphorylation of Akt and mTOR, as well as the expression of PI3K, which inhibit cardiomyocyte apoptosis and autophagy [54]. Finally, in diffuse large B-cell lymphoma cells, apigenin inhibits the activation of PI3K and proteins like mTOR, Akt, IKK, and p65 involved in the PI3K signal pathway [73].

#### 3.5.2. Apigenin and the MAPK/ERK Pathway

In addition to inhibiting the PI3K/Akt signaling pathway, apigenin was proven to modulate the MAPK/ERK pathway, another oncogenic pathway that plays an important role in cell-cycle regulation, metabolism, cell apoptosis, survival, and proliferation [74]. The hyperactivation and deregulation of this signaling pathway are frequently observed in cancer and lead to uncontrolled growth and increased resistance to apoptosis.

The pathway may be influenced by apigenin in various cancers, in vitro and in vivo [20]:

- In human melanoma cells, apigenin suppresses cell proliferation and cell migration along with the induction of apoptosis via decreasing the expression of phosphorylated (p)-ERK1/2 proteins [68];
- In colorectal cancer cells, apigenin enhances ABT-263-induced anti-tumor activity via the inhibition of Akt and ERK pathways [75];
- In an autochthonous mouse prostate cancer model, apigenin administration suppresses prostate cancer progression by decreasing IGF/IGFBP-3 and inhibiting p-Akt and p-ERK1/2 [50].

#### 3.5.3. Apigenin and the JAK/STAT Pathway

The JAK/STAT signaling pathway is constantly activated in various human carcinomas and promotes tumorigenesis and metastasis by promoting the expression of genes that encode antiapoptotic proteins, cell-cycle regulators, and angiogenic factors [20].

In the murine melanoma B16F10 cell line, apigenin effectively suppresses STAT3 phosphorylation, decreases STAT3 nuclear localization, and inhibits STAT3 transcriptional activity [76]. Apigenin also downregulates the STAT3 target genes MMP-2, MMP-9, VEGF, and Twist1, which are important for cell migration and invasion [76]. In human myeloid leukemia HL60 cells and erythroid leukemia TF1 cells, apigenin decreases the phosphorylation of JAK2 and STAT3 and decreases STAT5 in TF1 cells [60,62].

Additionally, in the human HER2-overexpressing breast cancer cell lines BT-474, SKBR3, and MDA-MB-453, apigenin triggers cell apoptosis by suppressing JAK/STAT3 signaling and decreasing nuclear translocation of STAT3 [60].

#### 3.5.4. Apigenin and the NF-κB Pathway

The NF-κB signaling pathway is generally considered an active factor in survival and proliferation. NF-κB inhibits cell death [20,77] via the activation of the following target genes:

- Pro-survival genes (Bcl-2, Bcl-xL, survivin, XIAP);
- Cell-cycle-related genes (cyclin D1);
- Growth factor, inflammatory cytokines, and tumor metastasis genes (COX-2).

Transcription factor NF-κB is activated in human cancers and is found to promote EMT and tumor migration through the activation of different mediators, such as basic fibroblast growth factor, IL-8, MMP-2, -3, and -9 [38,78].

Apigenin treatment inhibits NF-κB activation, both in vitro and in vivo. In a prostate TRAMP mouse model, apigenin administration significantly decreases prostate tumor volumes and completely abolishes cancer cell metastasis by inhibiting IKK activation, which in turn leads to the suppression of NF-κB activation [77]. Lastly, apigenin sensitizes non-small cell lung cancer cells to TRAIL-induced apoptosis and suppresses the translocation of NF-κB from the cytoplasm to the nucleus. The drug also blocks the degradation of IkBα, which further blocks the separation of IkBα from the NF-κB heterodimer [76].

#### 3.5.5. Apigenin and the Wnt/β-Catenin Pathway

The Wnt/β-catenin signaling pathway is a conserved signaling axis participating in diverse physiological processes, such as proliferation, differentiation, apoptosis, migration, invasion, and tissue emulation, enhancing DNA damage repair, facilitating transcriptional plasticity, and promoting immune evasion [79,80]. In osteosarcoma cells, apigenin inhibits proliferation and invasion by inactivating Wnt/β-catenin signaling, thereby suppressing cell proliferation, migration, and invasion [81].

Another study has shown that apigenin reduces the amount of total, cytoplasmic, and nuclear β-catenin, through the induction of the autophagy–lysosomal system. Furthermore, the auto-lysosomal degradation of β-catenin by apigenin occurs via the inhibition of the Akt/mTOR signaling pathway [82].

## 4. Combination Therapy for Apigenin

Chemotherapy drugs play a considerable and unavoidable role in the extension of the overall survival rates of cancer patients. However, their undesired toxicity remains a significant source of concern for both patients and clinicians. The integration of natural bioactive compounds, in specific cases, could potentiate anticancer efficacy and reduce the side effects of chemotherapy drugs [83].

Studies have shown that co-administration with apigenin significantly enhances the anticancer efficacy of chemotherapy drugs and helps to overcome their limitations in various types of cancers by targeting multiple signaling pathways [20,84]. The most common mechanisms apigenin uses to amplify the chemotherapy drugs’ efficacy are autophagy and apoptosis. Various mechanisms, such as cell-cycle regulation, tumor cell migration inhibition, invasion, and the stimulation of the immune response, can be responsible for chemo-sensitizing properties of apigenin in co-therapies [85].

Recent studies have evaluated the combination of apigenin with the following chemotherapy drugs: 5-FU, Cetuximab, Cisplatin, Cyclophosphamide, Doxorubicin, Gemcitabine, Paclitaxel, Sorafenib, Tamoxifen, Abivertinib, Gefitinib, Methotrexate, and Vincristine [86,87,88,89,90,91]. In all cases, synergic and/or additive effects were shown compared to monotherapy and there was a reduction in the side effects of monotherapies.

Furthermore, apigenin has been displayed to have significant chemo- and radio-protective properties [58,92]. Apigenin was found to have protective effects against doxorubicin and adriamycin-induced cardiotoxicity in vivo [93,94], and to prevent Cisplatin-induced nephrotoxicity by reducing the serum levels of TNF-α, IL-6, COXI, COXII, creatinine, and blood urea nitrogen, and increasing serum GSH levels [95]. As a radioprotector, apigenin has shown protective effects on human lymphocytes exposed to 137 Cs and to Cobalt 60 radiation. Apigenin pretreatment significantly reduced DNA damage and radiation-induced anomalies such as micronuclei, nucleoplasmic bridges, and nuclear buds in human peripheral-blood lymphocytes [58].

Furthermore, apigenin has shown protective effects against the UVA-induced senescence of normal human dermal fibroblasts and significantly inhibited UVB-induced carcinogenesis by promoting the expression of the antiangiogenic protein thrombospondin-1 in skin keratinocytes in vitro and in vivo [58].

## 5. Critical Aspects of Apigenin for Therapeutic Purposes

### 5.1. Bioavailability of Apigenin

A crucial aspect of a potential therapeutic drug is its bioavailability, the fraction of a drug reaching the systemic circulation, and the site of action where it can exert its biological effects. It is influenced by numerous factors, such as the substance’s chemical structure, the bond with other molecules (e.g., by acetylation or glycosylation), and intrinsic factors of the organism (e.g., the composition of the intestinal microbiota).

Apigenin has low bioavailability because of its low lipid (0.001–1.63 mg/mL) and water (2.16 μg/mL) solubility [37,96]. The bioavailability of apigenin also depends on its bioaccessibility, which refers to the extraction of apigenin from the food matrix during gastrointestinal digestion and the transformation into compounds available for absorption [97]. The degree and site of glycosylation influence gastrointestinal digestion: glycosides of apigenin survive acid hydrolysis in the stomach, and it goes to the duodenum unbroken. Further digestion and absorption depend on the nature of the modification and distribution of enzymes required to produce bioactive apigenin. Various cells can metabolize apigenin intracellularly through the enzymes present in the brush border epithelium, while the indigestible glycosides require extracellular de-glycosylation through bacterial enzymes present in the colon [98].

Apigenin taken orally is absorbed systemically with an availability of approximately 30% higher in the colon (40%) and lower in the terminal ileum (21%) via passive transport and independently of concentration. In vivo, in the duodenum and in the jejunum, apigenin was also mediated by active transport, as well as by the concentration-dependent membrane permeability whose highest level (maximum plasma concentration—Cmax) is, however, reached with a maximum plasma concentration time (Tmax) of 0.5–2.5 h. It should be remembered that the glycosylated forms of apigenin (e.g., 7-O-glucoside, 6-C-glucoside, or 8-C-glucoside) are metabolized by β-glucosidases in the stomach and small intestine to generate free apigenin (i.e., a form of aglycone). *Eubacterium ramulus* and *Bacteroides distasonis* have been identified as the main bacterial species essential for the biotransformation of 7-glycosides into aglycone apigenin [99,100].

Various strategies and techniques have been investigated to improve the bioavailability of apigenin:

- Water-in-oil-in-water (W/O/W) double emulsions loaded with apigenin. In vitro studies have confirmed the double emulsion’s capacity to transport bioactive compounds in an aqueous phase, minimizing degradation and potentially increasing in vivo bioavailability [101];
- Gold nanoparticles, widely used for their good biodistribution, stability, and low toxicity. Au3+ can be reduced by apigenin at a pH of 10 and at room temperature, forming highly stable and spherical apigenin-AuNPs. The apigenin-AuNPs are found to exhibit toxicity towards the A431 (epidermoid squamous cell carcinoma) cell line while being non-toxic towards normal epidermoid cells. This technique shows promise in the treatment of skin cancer [102];
- Phytosome, a phospholipid-based complex of apigenin, i.e., apigenin–phospholipid phytosome (APLC). Phytosome is highly compatible with human physiology and bioavailable thanks to its ability to cross the lipid bilayer membrane of enterocytes and reach systemic circulation. A study shows that APLC formulation demonstrated an over 36-fold higher aqueous solubility of apigenin, compared to that of pure apigenin [103];
- Self-microemulsifying drug-delivery systems (SMEDDSs). They are mixtures of oils, surfactants, solvents, and drug substances that form oil-in-water microemulsions with droplet sizes less than 100 nm when introduced into aqueous phases under gentle agitation or gastrointestinal motility [104]. A study shows that SMEDDSs could enhance the solubility and dissolution of apigenin and would be a potential carrier to improve the oral absorption of apigenin [105];
- Bioactive self-nanoemulsifying drug-delivery systems (BioSNEDDSs). They form a nanoemulsion with droplet sizes significantly smaller (by a factor of ten or similar) than droplets found in ordinary emulsions. The decreased droplet size increases the absorption rate and extent and prevents drug degradation in the gastrointestinal tract [106]. The BioSNEDDSs differ from conventional SNEDDSs for using bioactive lipid excipients such as black seed oil, *Moringa oleifera* seed oil, avocado oil, apricot oil, grape seed oil, safflower oil, and coconut oil fatty acid. A study shows that BioSNEDDSs formulated for apigenin provide collective advantages, such as a superior self-emulsification efficiency with an improved physical stability, high drug-loading capacity, antibacterial activity, and elevated apigenin bioavailability [107].

### 5.2. Absorption, Distribution, Metabolism, Excretion

Absorption, distribution, metabolism, and excretion are four processes that together describe a drug’s passage through the body.

#### 5.2.1. Absorption

Flavones are typically present in food as glycosides. The absorption of orally delivered apigenin has been the subject of animal studies, particularly in rats [108]. Most of these studies show that apigenin aglycones and O-glycosides are absorbed quickly: the Tmax is generally ≤ 1 h, with a Cmax of 1–100 mmol/L, depending on the dose and the food matrix [109]. Studies in humans that used celery leaves or parsley show plasma concentrations of <0.2 mmol/L with a Tmax > 7 h [110]. As for apigenin O-glycosides, rats are the most common animal model used for the absorption of apigenin C-glycoside. The Tmax for C-glycosides studied was <1 h and the Cmax was 1–29 mmol/L, varying with the dose. Depending on the sugar fraction, the absorption could occur in the small intestine or colon after deglycosylation. Glucosides are generally the only glycosides that can be absorbed from the small intestine and the absorption involves the glucose transport pathway. It is hypothesized that apigenin glucosides can be hydrolyzed by cytosolic *β*-glucosidase (CBG) and lactase-phlorizin hydrolase (LPH). The aglycone resulting after deglycosylation may then enter epithelial cells by passive diffusion. LPH is a membrane-bound enzyme found in the brush border of the small intestine and CBG is a broad-specificity cytosolic enzyme found in abundance in the liver, kidney, and small intestine of mammals. For CBG to cleave flavonoid glycosides, the molecules must first be actively or passively transported into the cytosol [110].

Although the sugar transporter SGLT1 may facilitate the absorption of certain glycosides, such as quercetin-3-glucoside and quercetin-4′-glucoside, into epithelial cells, it does not appear to transport flavones. In fact, apigenin and its glycosides (apigenin-6-C-glucoside, apigenin-7-O-glucoside, apigenin-8-O-glucoside) seem to be absorbed by epithelial cells of the small intestine only after hydrolyzation into aglycones. Flavonoids that cannot be absorbed from the small intestine, as well as the absorbed flavonoids secreted with bile, will be degraded in the colon by the microbiota [18].

#### 5.2.2. Distribution

Multiple in vivo studies have shown that apigenin has a good distribution to tissues, mainly the liver and intestine, due to its capacity to bind to soluble proteins in the blood and tissues. Wan et al. [111] showed that following the IV administration of 20 mg/kg of apigenin in rats, the initial blood concentration of apigenin (10 μg/mL) dropped rapidly within 30 min (100 ng/mL). Still, this rapid decline was followed by a slow disappearance of apigenin from the circulation, with a T ½ close to 8 h. Furthermore, the Vd of apigenin was much larger than the total body water of rats. In another study [112], mice were fed a diet containing apigenin for 5, 6, or 7 days. It was found that with a dose of 1.1 mmol/kg apigenin, the plasma levels of apigenin reached a steady state after 5 days with concentrations of apigenin in plasma, liver, and the small intestinal mucosa of 0.09 ± 0.08 nmol/mL, 1.5 ± 1.0 nmol/g, and 86 ± 47 nmol/g, respectively. Following the oral administration of *Chrysanthemum morifolium* extracts in rats [113], apigenin and luteolin were observed to be distributed along all the tissues of the gastrointestinal tract with concentrations three to ten times higher in the jejunum, as compared to other segments of the intestines.

The post-absorption tissue distribution of apigenin is driven by its compatibility with the human serum transferrin glycoprotein at the Fe3+ binding site using the interaction between the electrostatic and hydrogen bonds of the C5 and C7 hydroxyl groups of the apigenin B ring, the component amino acids Lys-291 and Tyr-188 of transferrin, and between the carbonyl of the C ring of apigenin and Arg-124 of transferrin. It is useful to remember that the distribution to the tissues as a consequence of the intrinsic lipophilicity is, for apigenin, supported by the LogP of 2.84 (the LogP or Log Kow measure of the differential of the solubility of chemical compounds in two solvents). Therefore, it is well below the LogP threshold > 5 (high lipophilicity) for a drug to be active orally and by passive diffusion to cross the membrane and bypass the blood–brain barrier, according to Lipinski’s rule [114].

#### 5.2.3. Metabolism

The absorbed apigenin may go through functionalization reactions (phase I metabolism) and conjugation reactions (phase II metabolism) in the intestine and liver [113,115]. Phase I reactions lead to the introduction of functional groups that give the molecule a particular chemical reactivity. In the presence of NADPH, these reactions are catalyzed by two families of liver enzymes: cytochromes and flavin-containing monooxygenase [45]. Phase II reactions lead to the formation of a covalent bond with endogenous molecules such as glucuronic acid, sulfate, glutathione, amino acids, or the acetate ion. These conjugated flavonoids may also be transported through the efflux transporters multi-drug resistance protein-1 (also referred to P-gp, ABCB1, CD-243) and multi-drug resistance-associated protein-2 (also referred to ABCC2 and CMOAT), whose distribution can be dramatically altered in cancers that regulate the intra-individual variability of the absorption in the intestinal tract [116].

Metabolites generated during phase I (luteolin) and phase II (sulfated and glucuronated conjugates) have four possible pathways [106]:

1. Direct systemic absorption;
2. Excretion;
3. Passage with the bile from the liver to the intestine, where they are hydrolyzed by bacterial beta-glucuronidases, returning to an absorbable form again (entero-hepatic circulation);
4. Passage into the intestinal lumen, where they are subject to hydrolysis and subsequently reabsorbed (entero-enteric circulation and local enteric circulation) [117].

#### 5.2.4. Excretion

Apigenin’s high volume of distribution and enterohepatic/enteric recycling processes indicate that the elimination patterns of this lipophilic molecule will be delayed [106]. A study [118] observed that, following a single oral administration of radio-labeled apigenin, approximately 50% of apigenin was recovered in urine and 12% in feces. Although most products were excreted within the first 24 h, about 25% of the original apigenin dose was retained 10 days after treatment, suggesting apigenin’s slow absorption and elimination in the body [115].

## 6. Conclusions

Throughout centuries, botanical and nutritional compounds have been used to treat and prevent diseases, thereby creating knowledge of the potential of natural agents, known as phytochemicals, as anticarcinogenic agents [9,10,119]. Among natural compounds, polyphenols, the most important of which include flavonoids, display many anticarcinogenic properties [120].

Apigenin-containing plant preparations have been used as traditional medicines for their anti-diabetic properties [44] and to treat diseases with an inflammatory and/or degenerative component [42,69,121]. In the 1980s, apigenin was proposed to interfere with the process of carcinogenesis and successive studies have suggested that it can be a therapeutic agent against different types of cancers, due to its properties in modulating different hallmarks of cancer and stimulating an immune response [4,6]. Apigenin has been reported to target multiple signaling pathways, such as PI3K/Akt/mTOR, MAPK/ERK, JAK/STAT, NF-kB, and Wnt/β-catenin pathways, involved in the development and progression of cancer.

Combining natural bioactive compounds with chemotherapy drugs can potentiate anticancer efficacy and reduce the side effects [83]. Studies have shown that co-administration with apigenin significantly enhances the anticancer efficacy of chemotherapy and helps to overcome its limitations in various types of cancers by targeting multiple signaling pathways. The most common mechanisms used by apigenin to amplify the chemotherapy efficacy are autophagy and apoptosis [43,62]. Various mechanisms, such as the regulation of the cell cycle, inhibition of tumor cell migration, invasion, and stimulation of the immune response can be responsible for chemo-sensitizing properties of apigenin in co-therapies.

Several investigations have shown that a number of chemotherapy drugs [86,87,88,89,90,91] show synergic and/or additive effects and a reduction of side effects when combined with apigenin, compared to monotherapy [83,122].

Hence, it is emerging that apigenin can represent a potentially useful tool in the fight against cancer. Its low bioavailability due to low lipid and water solubility, which could mean criticism of apigenin’s clinical applicability, can be improved by various strategies and techniques, as reported above. The studies herein reviewed indicate that apigenin is one of the most fascinating natural compounds to be used in combination with different antiblastic treatments. Consequently, we strongly suggest considering these results when designing combined treatment protocols for humans based on new formulations with low toxicity, easy handling, and the ability to reduce side effects without interfering with the antiblastic action mechanism.

## Data Availability

Data sharing not applicable.

## Supplementary Materials

The supporting information can be downloaded at: https://www.mdpi.com/article/10.3390/ijms25010251/s1.
