# Supplementary material for: Resources for Human Health from the Plant Kingdom: The Potential Role of the Flavonoid Apigenin in Cancer Counteraction
Source: Int J Mol Sci. 2023 Dec 23;25(1):251. doi: 10.3390/ijms25010251 (PMC10778966; doi:10.3390/ijms25010251)
Supplement: Supplementary file 1 [file ijms-25-00251-s001.zip › ijms-2771242-supplementary.pdf]

Table S1. Natural sources of apigenin

Natural sources of apigenin	
Artichoke	*Cynara cardunculus*
Assai palm	*Euterpe oleracea Mart.*
Barbed skullcap	*Scutellaria barbata*
Bellyache bush	*Jatropha gossypifolia*
Celeriac	*Apium graveolens*
Celery	*Apium graveolens*
Chamomile	*Matricaria chamomilla*
Chinese celery	*Apium graveolens* var. *secalinum*
Chinese daphne	*Daphne genkwa*
Chinese wedelia	*Wedelia chinensis*
Common clubmoss	*Lycopodium clavatum*
Creat	*Andrographis paniculata*
Florist's daisy	*Chrysanthemum morifolium*
Ginkgo	*Ginkgo biloba*
Horsemint	*Mentha longifolia*
Juniper berries	*Juniperus communis*
Korean perilla	*Perilla frutescens*
Kumquat	*Fortunella* spp
Maize	*Zea mays*
Onions	*Allium cepa*
Oregano	*Origanum vulgare*
Parsley	*Petroselinum crispum*
Peppermint	*Mentha piperita*
Rosemary	*Rosmarinus officinalis*
Sage	*Salvia officinalis*
St John's wort	*Hypericum perforatum*
Sweet acacia	*Acacia farnesiana*
Thyme	*Thymus vulgaris*
Water horsetail	*Equisetum fluviatile*
Wheat sprouts	*Triticum aestivum*
White / Red sorgum	*Sorghum bicolour*
